# Selection of the promising fig (*Ficus carica* L.) accessions using fruit‐related characters

**DOI:** 10.1002/fsn3.2886

**Published:** 2022-04-15

**Authors:** Ali Khadivi, Farhad Mirheidari

**Affiliations:** ^1^ 125649 Department of Horticultural Sciences Faculty of Agriculture and Natural Resources Arak University Arak Iran

**Keywords:** breeding, conservation, fruit color, fruit quality, wild edible fig

## Abstract

Nowadays, fig (*Ficus carica* L.) fruits are consumed either fresh or dried and used for jam or spirit beverage production. Morphological and pomological diversity of 49 wild edible fig accessions sampled was evaluated. Analysis of variance revealed significant differences among the wild accessions studied using the morphological data recorded. Ripening time ranged from late July to mid‐August. Fruit skin ground color showed high variability, including cream–purple (4 accessions), purple–yellow (8), light purple (7), purple (15), dark purple (5), purple–cream (3), and cream (7). The range of fruit‐related traits was as follows: fruit length: 12.65–22.60 mm, fruit width: 10.67–24.18 mm, fruit fresh weight: 2.52–6.13 g, and fruit flesh thickness: 0.85–1.89 mm. Principal component analysis (PCA) showed 10 independent components that could explain 84.11% of total variance. Ward dendrogram created according to the data obtained revealed the variation among the accessions and showed two major clusters. The present results showed that the studied accessions had remarkable phenotypic variation, and among them, some accessions with high‐quality fruits in size, color, and taste can be planted and then used in the breeding programs. Information on the current levels of genetic diversity of germplasm is essential for devising strategies for wild forms conservation.

## Graphical abstract

The studied fig (*Ficus carica* L.) accessions had remarkable phenotypic variation, and among them, some accessions with high‐quality fruits in size, color, and taste can be planted and then used in the breeding programs. Information on the current levels of genetic diversity of germplasm is essential for devising strategies for wild forms conservation.

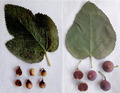

## 1 INTRODUCTION

Fig tree (*Ficus carica* L., 2*n* = 26) is native to the Western Asia and later migrated to the Mediterranean areas. Kislev et al. (2006) reported that figs could be the first domesticated tree of the Neolithic Revolution, which occurred a thousand years before cereals. It is reported that the fig plant was domesticated 5000 years before millet or wheat. Given this historical background, the evaluation and discovery of the genetic diversity of figs has been considered by scientists (Hirst, 1996). Fig tree breeding programs have not expanded much; therefore, its different populations show considerable diversity. Also, there are about 600 local cultivars of figs known as varieties (Condit, 1955). These varieties are propagated by cuttings and there are selections of them that are used for edible fruits. The nature of fig trees is such that it is well adapted to dry weather with hot summers, which has led to the development of large roots in this plant to be able to absorb water from the soil and from distances away from the trunk. Such thermophilic trees are suitable for adaptation to consequences of the climate change and global warming, and thus allow fig trees to be grown in hot and dry areas where other species are not viable (Sugiura et al., 2007).

Today, figs are consumed both fresh and dried, and are used to produce jams or spirits. The fig tree is the oldest fruit tree known as gynodioecious and is pollinated by the bee *Blastophaga psenes* L. (Kislev et al., 2006). Figs have ecotypes known as common figs (unisexual ion female trees) and caprifigs (bisexual with functional male trees) and have similar frequencies in wild populations (Valdeyron & Lloyd, 1979).

Food production and security is highly dependent on the responsible use and protection of agrodiversity and gene pool. On‐farm conservation has been encouraged among international initiatives to preserve the genetic diversity of local and traditional varieties (Esquinas‐Alcazar, 2005). Collecting, conserving, and evaluating the genetic diversity of fig accessions can help to establish breeding programs and improve the studied landraces, and thus identify and introduce the selections that have disease resistance as well as good fruit quality and yield. Wild figs are distributed in the Middle East and the Mediterranean areas (De Candolle, 1885). According to valid botanical documents, Southern Arabia, Turkey, Iran, and Syria are the most important sources of edible figs (Flaishman et al., 2008; Giraldo et al., 2005).

Iran has rich resources of different plant species. Investigating the genetic diversity of wild accessions and identifying native varieties in each region is the first step to conserving genetic resources. Genetic erosion is one of the most serious threats to germplasm extinction. Therefore, there is little knowledge about ancient landraces (Mars et al., 2008). To protect the genetic resources of figs, several alternative conservation strategies have been considered. Morpho‐pomological characterizations are the main basis for selection and identification of genotypes as well as evaluation of genetic diversity in figs (Oukabli et al., 2002). Morphological characterizations, including leaf‐ and fruit‐related traits are used to better distinguish between wild and cultivated plants (Asanidzea et al., 2011). In the present study, the morpho‐pomological traits were investigated to select superior wild accessions of fig crop.

## 2 MATERIAL AND METHODS

### 2.1 Plant material

Morphological and pomological diversity of 49 wild edible fig (*F. carica*) accessions sampled from natural habitats of Jasb region in Isfahan province, Iran was evaluated in summer of 2021. Jasb region is located at 34°02′54″N latitude, 50°45′28″E longitude, and 1785 m height above sea level. The appropriate distances were considered between the accessions to avoid the possibility of sampling and collecting clones of the selected trees.

### 2.2 The characters evaluated

A total of 55 quantitative and qualitative morphological and pomological traits (Table 1) were used for phenotypic evaluations according to the guidelines provided by the International Plant Genetic Resources Institute (IPGRI, 2003) in the fig descriptors. Fifty replicates for leaves and fruits were used for measurements and the mean values were taken for analysis. Dimensions of leaf, fruit, and seed were measured using a digital caliper. Fruit fresh weight was measured using an electronic balance with 0.01 g precision. The rest characters (Table 2) were qualitatively estimated based on rating and coding according to the fig descriptors (IPGRI, 2003).

**TABLE 1:** Statistical descriptive parameters for morphological traits used to study the wild *F. carica* accessions

No.	Trait	Unit	Min.	Max.	Mean	SD	CV (%)
1	Tree growth habit	Code	1	9	3.53	2.30	65.18
2	Tree vigor	Code	1	5	2.59	1.41	54.56
3	Trunk color	Code	1	5	2.96	1.66	56.01
4	Shoot color	Code	1	9	4.31	2.10	48.82
5	Tree height	Code	1	5	2.55	1.43	56.08
6	Branching	Code	1	5	3.08	1.47	47.73
7	Branch density	Code	1	5	3.37	1.33	39.58
8	Branch flexibility	Code	1	5	2.63	1.27	48.29
9	Trunk type	Code	1	5	2.76	1.56	56.59
10	Trunk diameter	Code	1	5	2.35	1.44	61.15
11	Canopy density	Code	1	5	3.69	1.45	39.19
12	Tendency to form suckers	Code	1	5	3.41	1.58	46.33
13	Leaf density	Code	1	5	3.65	1.32	36.05
14	Leaf shape	Code	1	3	2.43	0.91	37.57
15	Leaf base shape (petiole sinus)	Code	1	5	3.00	0.71	23.57
16	Leaf length	mm	50.23	156.81	106.81	21.83	20.44
17	Leaf width	mm	33.28	110.98	78.44	15.07	19.21
18	Leaf color	Code	1	3	1.57	0.91	58.15
19	Leaf venation clarity	Code	1	5	2.96	1.50	50.64
20	Main leaf venation number	Number	1	3	1.57	0.91	58.15
21	Lobe number	Number	0	3	0.86	1.37	159.19
22	Central lobe length	mm	0.00	71.50	17.19	27.75	161.42
23	Lateral lobe depth	mm	0.00	13.68	2.04	3.93	192.50
24	Lateral lobe vein length	mm	0.00	77.51	18.70	30.05	160.68
25	Lateral lobe base width	mm	0.00	26.50	6.33	10.21	161.23
26	Leaf margin	Code	1	11	6.76	2.99	44.23
27	Hair density on leaf upper surface	Code	1	5	2.76	1.67	60.33
28	Hair density on leaf lower surface	Code	1	5	1.98	1.30	65.61
29	Petiole length	mm	13.26	48.75	28.67	7.77	27.09
30	Petiole thickness	mm	1.51	3.74	2.83	0.49	17.46
31	Petiole color	Code	1	3	1.16	0.55	47.67
32	Ripening time	Code	1	5	2.43	1.41	58.19
33	Fruit yield	Code	1	5	3.08	1.35	43.86
34	Fruit length	mm	12.65	22.60	16.76	2.91	17.37
35	Fruit width	mm	10.67	24.18	16.18	3.43	21.18
36	Fruit fresh weight	g	2.52	6.13	3.47	1.03	29.71
37	Fruit shape	Code	1	5	2.76	1.27	45.91
38	Fruit symmetry	Code	0	1	0.90	0.31	34.00
39	Fruit base shape	Code	1	3	2.43	0.91	37.57
40	Fruit apex shape (near ostiole)	Code	1	3	1.94	1.01	51.96
41	Ostiole form	Code	1	3	2.43	0.91	37.57
42	Ostiole width	mm	1.44	4.27	2.39	0.52	21.67
43	Fruit stalk shape	Code	1	5	4.55	1.24	27.32
44	Fruit stalk length	mm	2.78	21.11	9.90	4.33	43.77
45	Fruit stalk width	mm	1.41	3.45	2.25	0.49	21.82
46	Fruit neck length	mm	2.45	13.33	4.99	2.28	45.59
47	Fruit skin ground color	Code	1	13	6.88	3.59	52.21
48	Fruit flesh color	Code	1	7	3.78	1.91	50.45
49	Internal pulp color	Code	1	7	2.76	1.71	62.10
50	Fruit flesh thickness	mm	0.85	1.89	1.40	0.28	20.21
51	Fruit quality	Code	1	5	3.20	1.37	42.78
52	Seed amount	Code	1	5	3.12	1.50	47.92
53	Seed length	mm	1.12	2.14	1.63	0.20	12.21
54	Seed width	mm	0.78	1.63	1.15	0.16	13.74
55	Seed thickness	mm	0.76	1.35	0.91	0.14	15.27

**TABLE 2:** Frequency distribution for the measured qualitative morphological characters in the wild *F. carica* accessions studied

Trait	Frequency (no. of accessions)							
	0	1	3	5	7	9	11	13
Tree growth habit	‐	Weeping (15)	Spreading (16)	Open (10)	Semierect (6)	Erect (2)	‐	‐
Tree vigor	‐	Low (18)	Intermediate (23)	High (8)	‐	‐	‐	‐
Trunk color	‐	Gray–white (17)	Gray (16)	Gray–Brown (16)	‐	‐	‐	‐
Shoot color	‐	Brown (7)	Dark brown (14)	Gray–white (20)	Gray (5)	Gray–Brown (3)	‐	‐
Tree height	‐	Small (to 1.5 m) (19)	Intermediate (1.5–3 m) (22)	Tall (3 m and more) (8)	‐	‐	‐	‐
Branching	‐	Low (12)	Intermediate (23)	High (14)	‐	‐	‐	‐
Branch density	‐	Low (7)	Intermediate (26)	High (16)	‐	‐	‐	‐
Branch flexibility	‐	Low (15)	Intermediate (28)	High (6)	‐	‐	‐	‐
Trunk type	‐	Multitrunk/Low (18)	Multi/Intermediate (19)	Multi/High (12)	‐	‐	‐	‐
Trunk diameter	‐	Low (23)	Intermediate (19)	High (7)	‐	‐	‐	‐
Canopy density	‐	Low (7)	Intermediate (18)	High (24)	‐	‐	‐	‐
Tendency to form suckers	‐	Low (11)	Intermediate (17)	High (21)	‐	‐	‐	‐
Leaf density	‐	Low (5)	Intermediate (23)	High (21)	‐	‐	‐	‐
Leaf shape	‐	Base decurrent (14)	Leaf not lobed (35)	‐	‐	‐	‐	‐
Leaf base shape (petiole sinus)	‐	Truncate (3)	symmetric cordate (43)	Asymmetric cordate (3)	‐	‐	‐	‐
Leaf color	‐	Green (35)	Dark green (14)	‐	‐	‐	‐	‐
Leaf venation clarity	‐	Low (14)	Intermediate (22)	High (13)	‐	‐	‐	‐
Lobe number	Absent (35)	Present (14)	‐	‐	‐	‐	‐	‐
Leaf margin	‐	Sinuate (3)	Broad sinuate (7)	Dentate (11)	Broad denate (7)	Serrate (14)	Broad serrate (7)	‐
Hair density on leaf upper surface	‐	Low (20)	Intermediate (15)	High (14)	‐	‐	‐	‐
Hair density on leaf lower surface	‐	Low (29)	Intermediate (16)	High (4)	‐	‐	‐	‐
Petiole color	‐	Light green (45)	Green–light brown (4)		‐	‐	‐	‐
Ripening time	‐	Late July (21)	Early August (21)	Mid‐August (7)	‐	‐	‐	‐
Fruit yield	‐	Low (10)	Intermediate (27)	High (12)	‐	‐	‐	‐
Fruit shape	‐	Oblong (13)	Globose (29)	Oblate (7)	‐	‐	‐	‐
Fruit symmetry	No (5)	Yes (44)	‐	‐	‐	‐	‐	‐
Fruit base shape	‐	Rounded (14)	Acute (subconical) (35)	‐	‐	‐	‐	‐
Fruit apex shape (near ostiole)	‐	Rounded (26)	Flat (23)	‐	‐	‐	‐	‐
Ostiole form	‐	Flat (14)	Embossed (35)	‐	‐	‐	‐	‐
Fruit stalk shape	‐	Variously enlarged/C (5)	Long and slender/G (1)	Long and slender/H (43)	‐	‐	‐	‐
Fruit skin ground color	‐	Cream–purple (4)	Purple–yellow (8)	Light purple (7)	Purple (15)	Dark purple (5)	Purple–cream (3)	Cream (7)
Fruit flesh color	‐	White (6)	Cream (28)	Light red (5)	Light violet (10)	‐	‐	‐
Internal pulp color	‐	Red (19)	Light brown (19)	Brown (9)	Dark brown (2)	‐	‐	‐
Fruit quality	‐	Low (9)	Intermediate (26)	High (14)	‐	‐	‐	‐
Seed amount	‐	Low (12)	Intermediate (22)	High (15)	‐	‐	‐	‐

### 2.3 Statistical analysis

Analysis of variance (ANOVA) was performed to evaluate the variation among accessions based on the traits measured using SAS software (SAS Institute, 1990). Simple correlations between traits were determined using Spearman correlation coefficients (SPSS Inc., Norusis, 1998). Principal component analysis (PCA) was used to investigate the relationship between the accessions and determine the main traits effective in accession segregation using SPSS software. Hierarchical cluster analysis (HCA) was performed using Ward's method and Euclidean coefficient using PAST software (Hammer et al., 2001). The first and second principal components (PC1/PC2) were used to create a scatter plot with PAST software.

## 3 RESULTS AND DISCUSSION

The ANOVA revealed significant differences among the wild accessions studied using the morphological data recorded. Seed length exhibited the lowest CV (12.21%) and followed by seed width (13.74%), seed thickness (15.27%), fruit length (17.37%), petiole thickness (17.46%), and leaf width (19.21%). The rest 49 characters had the CV more than 20.00%, indicating strong diversity among the accessions, so that CV in 21 traits was more than 50.00%. Lateral lobe depth showed the highest CV (192.50%) and followed by central lobe length (161.42%), lateral lobe base width (161.23%), lateral vein length (160.68%), and lobe number (159.19%) (Table 1).

Tree growth habit was highly variable and included weeping (15 accessions), spreading (16), open (10), semierect (6), and erect (2). The intermediate rating was predominant for tree vigor, tree height, branching, branch density, branch flexibility, leaf density, and leaf venation clarity. Leaf shape was base decurrent (14 accessions, having lobe) and not lobed (35) (Table 2). In those 14 accessions with having lobe in leaf, lobe number was 3, and the range of lobe‐related traits was as follows: central lobe length: 50.88–71.50 mm, lateral lobe depth: 3.01–13.68 mm, lateral lobe vein length: 58.20–77.51 mm, and lateral lobe base width: 17.35–26.50 mm (Table 1).

Leaf base shape (petiole sinus) was predominantly symmetric cordate (43 accessions). Leaf margin showed strong diversity, including sinuate (3 accessions), broad sinuate (7), dentate (11), broad denate (7), serrate (14), and broad serrate (7). Hair density on leaf upper and lower surfaces was low in most of the accessions (Table 1). Leaf length ranged from 50.23 to 156.81 mm, and leaf width varied from 33.28 to 110.98 mm. The range of main leaf venation number was 1–3. The range of petiole length and width was 13.26–48.75 mm and 1.51–3.74 mm, respectively (Table 2).

Ripening time ranged from late July to mid‐August. Fruit yield was low in 10, intermediate in 27, and high in 12 accessions. Fruit base shape was acute (subconical) in most of the accessions (35). Fruit shape was predominantly globose (29). Aljane et al. (2008) indicated that the fruit shape is an important factor for packing and transportation. The globose shape is more dominant character (Caliskan & Polat, 2012; Gozlekci, 2011; Hssaini et al., 2020) and considered most suitable for efficient packing and fruits transportation (Benettayeb et al., 2017).

Fruit was symmetric in the majority of accessions (44). Fruit skin ground color showed high variability, including cream–purple (4 accessions), purple–yellow (8), light purple (7), purple (15), dark purple (5), purple–cream (3), and cream (7). Fruit flesh color was white (6 accessions), cream (28), light red (5), and light violet (10), while internal pulp color was red (19 accessions), light brown (19), brown (9), and dark brown (2) (Table 2). Skin color is an essential parameter, which affects consumer perception of fresh figs and used to determine their ripening period.

Fruit quality was low in 9, intermediate in 26, and high in 14 accessions. The range of fruit‐related traits was as follows: fruit length: 12.65–22.60 mm, fruit width: 10.67–24.18 mm, fruit fresh weight: 2.52–6.13 g, and fruit flesh thickness: 0.85–1.89 mm (Table 1). The range of fruit stalk length was 2.78–21.11 mm, fruit stalk width was 1.41–3.45 mm, and fruit neck length was 2.45–13.33 mm. The presence of a neck in figs facilitates picking the fruit from the tree, and is thus associated with easier harvesting (Trad et al., 2012). Short neck length is reported to cause fig fruit damages during harvest (Darjazi, 2011; Gozlekci, 2011).

**FIGURE 1:**
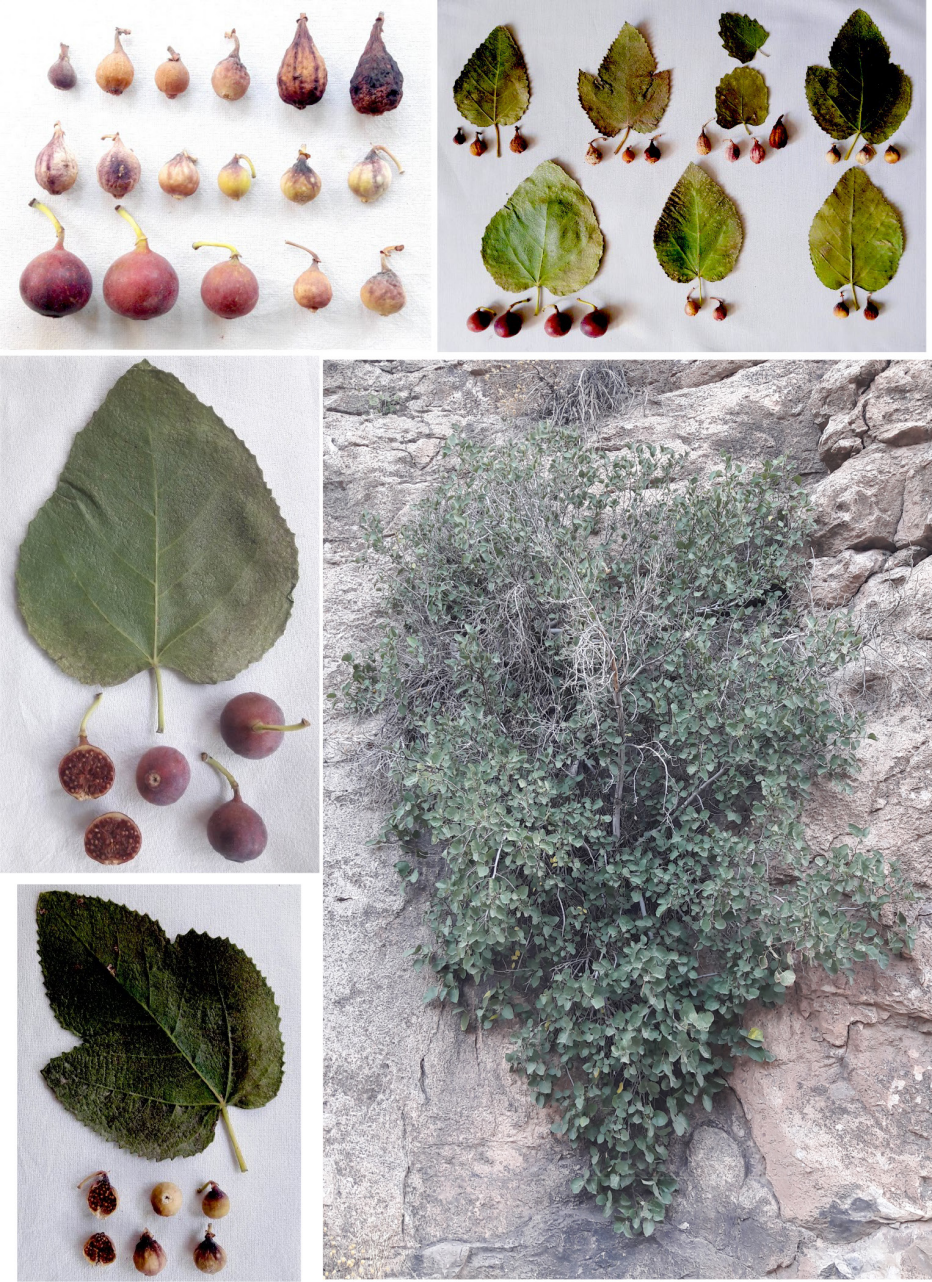
The pictures of leaves and fruits of the wild *F. carica* accessions studied

Ostiole form was embossed in most of the accessions (35). Ostiole width ranged from 1.44 to 4.27 mm. It is important to note that a large ostiole in the fig is an undesirable characteristic. The smaller the ostiole width, the better the fruit that can be stored and protected from infectious agents (Trad et al., 2012). Seed amount was low (12), intermediate (22), and high (15). The range of seed‐related traits was as follows: seed length: 1.12–2.14 mm, seed width: 0.78–1.63 mm, and seed thickness: 0.76–1.35 mm. Duman et al. (2018) reported the average of 1.13 mm for seed length and 1.00 mm for seed width in a fig collection from Turkey. The pictures of leaves and fruits of the wild *F. carica* accessions studied are shown in Figure 1.

Spearman correlation analysis showed significant correlation between some characters (data not shown). Leaf length showed positive correlations with leaf width (*r* = 0.75), petiole length (*r* = 0.52), petiole thickness (*r* = 0.48), lobe number (*r* = 0.51), central lobe depth (*r* = 0.53), and lateral lobe depth (*r* = 0.60), in agreement with previous studies in edible and caprifig figs (Anjam et al., 2017; Khadivi‐Khub & Anjam, 2014, 2016; Mirheidari et al., 2020). Fruit weight was positively correlated with leaf density (*r* = 0.44), leaf length (*r* = 0.41), leaf width (*r* = 0.55), leaf color (*r* = 0.42), fruit length (*r* = 0.62), and fruit width (*r* = 0.78), in agreement with previous studies in edible and caprifig figs (Anjam et al., 2017; Khadivi‐Khub & Anjam, 2014, 2016; Mirheidari et al., 2020).

The PCA showed 10 independent components that could explain 84.11% of total variance (Table 3). The PC1 accounted for 19.93% of total variance and included main leaf venation number (0.99), lobe number (0.99), central lobe length (0.98), lateral lobe depth (0.81), lateral lobe vein length (0.98), lateral lobe base width (0.98), hair density on leaf upper surface (0.87), hair density on leaf lower surface (0.83), and ostiole width (−0.58). Eight traits, including trunk color (−0.65), ripening time (0.93), fruit width (0.63), fruit fresh weight (0.82), fruit base shape (−0.79), fruit apex shape (near ostiole) (−0.69), ostiole form (−0.79), and fruit quality (0.72), loaded on PC2 and accounted for 13.32% of total variance. The PC3 was correlated with tree vigor(0.62), shoot color (−0.61), shoot color (−0.61), leaf length (0.82), leaf width (0.79), petiole length (0.56), petiole thickness (0.85), and fruit skin ground color (−0.57) that accounted for 11.13% of total variance. Generally, these results were in accordance with those reported in previous edible fig morphological studies (Ciarmiello et al., 2015; Darjazi, 2011; Gaaliche et al., 2011; Hssaini et al., 2020; Khadivi et al., 2018; Mars et al., 1998; Saddoud et al., 2011). They reported the importance of pomological characterization as main factor in discriminating and assessing breeding materials of fig trees. Caliskan et al. (2017) and Khadivi et al. (2018) reported that the pomological characteristics are important to evaluate the variation in traits of edible fig accessions. Furthermore, the selection of highly discriminant variables is important to optimize resources for a feasible morphological assessment.

**TABLE 3:** Eigenvalues of the principal component axes from the PCA of the morphological characters in the wild *F. carica* accessions studied

Trait	Components									
	1	2	3	4	5	6	7	8	9	10
Tree growth habit	0.40	−0.34	−0.03	0.23	0.61**	−0.07	0.09	−0.41	−0.07	−0.10
Tree vigor	0.02	0.13	0.62**	0.28	0.13	−0.04	0.06	−0.35	−0.21	−0.14
Trunk color	−0.04	−0.65**	0.14	0.44	0.13	0.08	−0.36	0.13	−0.07	0.07
Shoot color	−0.40	−0.06	−0.61**	−0.26	−0.24	0.21	−0.23	0.15	−0.03	−0.11
Tree height	−0.41	−0.03	0.21	−0.36	0.09	−0.30	0.30	−0.52	−0.02	−0.09
Branching	−0.14	−0.22	−0.19	0.71**	0.16	−0.04	0.30	−0.06	0.26	−0.23
Branch density	−0.01	−0.29	0.17	0.62**	0.16	0.25	−0.04	−0.10	0.33	−0.16
Branch flexibility	−0.01	0.02	0.60**	0.30	0.03	−0.07	0.14	0.15	−0.26	0.31
Trunk type	−0.01	−0.11	−0.05	0.75**	0.04	−0.06	0.16	0.20	0.01	−0.29
Trunk diameter	−0.26	0.13	0.07	0.15	−0.16	0.18	0.13	−0.59**	0.26	−0.12
Canopy density	−0.27	−0.15	0.06	0.79**	0.03	0.19	−0.06	0.06	0.13	0.13
Tendency to form suckers	−0.07	−0.08	−0.30	0.54	0.55**	−0.33	0.10	0.07	0.06	0.13
Leaf density	0.01	−0.16	0.21	0.78**	−0.01	0.07	0.04	−0.04	−0.15	0.06
Leaf shape	−0.99**	0.03	−0.09	0.01	−0.01	−0.04	0.05	−0.06	−0.01	0.02
Leaf base shape (petiole sinus)	0.00	0.13	0.31	−0.19	0.06	−0.01	0.15	−0.05	0.03	0.80**
Leaf length	0.03	0.10	0.82**	−0.23	−0.07	−0.25	0.14	0.02	0.09	0.13
Leaf width	0.31	0.38	0.79**	−0.07	−0.03	0.01	0.10	0.14	0.09	0.04
Leaf color	0.00	0.48	0.19	0.49	0.29	0.24	−0.03	−0.04	−0.26	−0.07
Leaf venation clarity	−0.36	−0.15	0.43	−0.47	−0.45	0.12	−0.11	0.10	0.23	−0.05
Main leaf venation number	0.99**	−0.03	0.09	−0.01	0.01	0.04	−0.05	0.06	0.01	−0.02
Lobe number	0.99**	−0.03	0.09	−0.01	0.01	0.04	−0.05	0.06	0.01	−0.02
Central lobe length	0.98**	−0.01	0.06	−0.04	−0.05	0.05	−0.03	0.07	−0.02	−0.02
Lateral lobe depth	0.81**	−0.21	0.27	0.29	0.34	0.01	−0.10	−0.03	−0.08	0.00
Lateral lobe vein length	0.98**	−0.05	0.12	0.03	0.06	0.04	−0.06	0.03	0.03	−0.02
Lateral lobe base width	0.98**	−0.02	0.06	−0.04	−0.05	0.03	−0.04	0.08	0.01	−0.03
Leaf margin	0.50	0.34	−0.33	0.45	0.00	0.40	−0.11	0.05	−0.05	−0.28
Hair density on leaf upper surface	0.87**	0.06	0.17	−0.15	0.11	−0.27	0.09	0.07	−0.03	0.09
Hair density on leaf lower surface	0.83**	0.00	−0.05	−0.20	0.15	−0.14	0.02	0.07	−0.13	0.08
Petiole length	0.49	−0.11	0.56**	0.18	0.28	−0.18	−0.25	−0.02	0.22	0.10
Petiole thickness	0.23	−0.08	0.85**	0.10	0.05	−0.23	−0.08	−0.07	−0.01	0.04
Petiole color	−0.20	−0.05	−0.11	−0.44	0.45	−0.47	0.03	0.09	−0.11	−0.20
Ripening time	−0.15	0.93**	−0.11	−0.20	0.09	0.05	0.00	−0.03	−0.11	−0.05
Fruit yield	−0.12	0.49	−0.22	0.08	−0.35	0.23	0.37	−0.09	−0.28	0.07
Fruit length	−0.40	0.51	0.26	0.29	−0.15	0.02	0.54	−0.18	0.05	0.07
Fruit width	−0.40	0.63**	0.42	−0.02	0.05	0.05	0.47	0.04	0.02	0.10
Fruit fresh weight	−0.06	0.82**	0.47	0.04	0.05	0.23	−0.05	−0.01	−0.01	0.11
Fruit shape	−0.23	0.44	0.41	−0.43	0.22	0.17	0.04	0.36	0.00	0.01
Fruit symmetry	0.17	−0.07	0.07	0.18	−0.09	0.15	0.06	0.79**	0.07	−0.17
Fruit base shape	−0.30	−0.79**	−0.20	0.31	0.29	−0.19	0.04	−0.08	−0.08	−0.07
Fruit apex shape (near ostiole)	−0.04	−0.69**	0.20	0.04	0.41	0.32	−0.05	0.10	0.02	−0.32
Ostiole form	−0.30	−0.79**	−0.20	0.31	0.29	−0.19	0.04	−0.08	−0.08	−0.07
Ostiole width	−0.58**	0.40	−0.08	0.02	0.33	0.10	0.41	0.10	−0.08	−0.17
Fruit stalk shape	−0.08	0.15	−0.01	0.15	−0.22	0.22	−0.10	−0.05	0.78**	0.05
Fruit stalk length	−0.33	0.50	0.18	−0.11	−0.40	0.42	−0.27	0.14	0.06	0.20
Fruit stalk width	0.06	−0.14	0.03	0.15	0.32	−0.27	0.82**	−0.04	−0.09	0.14
Fruit neck length	0.19	−0.36	0.20	0.21	0.61**	−0.06	0.21	0.00	−0.09	0.22
Fruit skin ground color	−0.23	0.36	−0.57**	0.26	0.02	−0.11	0.37	−0.15	−0.09	−0.22
Fruit flesh color	−0.45	0.09	0.29	0.07	−0.41	−0.01	0.60**	−0.03	−0.12	0.15
Internal pulp color	−0.36	−0.46	−0.41	0.04	0.20	−0.04	0.05	−0.25	−0.36	0.05
Fruit flesh thickness	0.02	0.12	0.32	−0.02	0.82**	−0.04	−0.06	0.08	−0.19	0.07
Fruit quality	−0.21	0.72**	−0.06	−0.04	0.13	−0.09	−0.06	−0.13	0.20	−0.04
Seed amount	−0.16	0.11	0.25	−0.35	−0.51	0.38	−0.05	0.25	−0.02	0.20
Seed length	−0.05	0.11	−0.16	−0.02	−0.14	0.84**	−0.15	0.16	0.22	−0.05
Seed width	0.08	−0.05	−0.21	0.10	−0.11	0.90**	−0.03	0.02	0.05	0.01
Seed thickness	−0.22	0.32	−0.36	0.20	0.10	0.67**	0.06	−0.06	−0.07	−0.06
Total	10.96	7.32	6.12	5.65	4.12	3.87	2.79	2.15	1.65	1.62
% of Variance	19.93	13.32	11.13	10.27	7.49	7.04	5.08	3.91	3.00	2.94
Cumulative %	19.93	33.25	44.38	54.65	62.14	69.18	74.26	78.17	81.16	84.11

Note

**Eigenvalues ≥0.55 are significant at the *p* ≤ .01 level.

**FIGURE 2:**
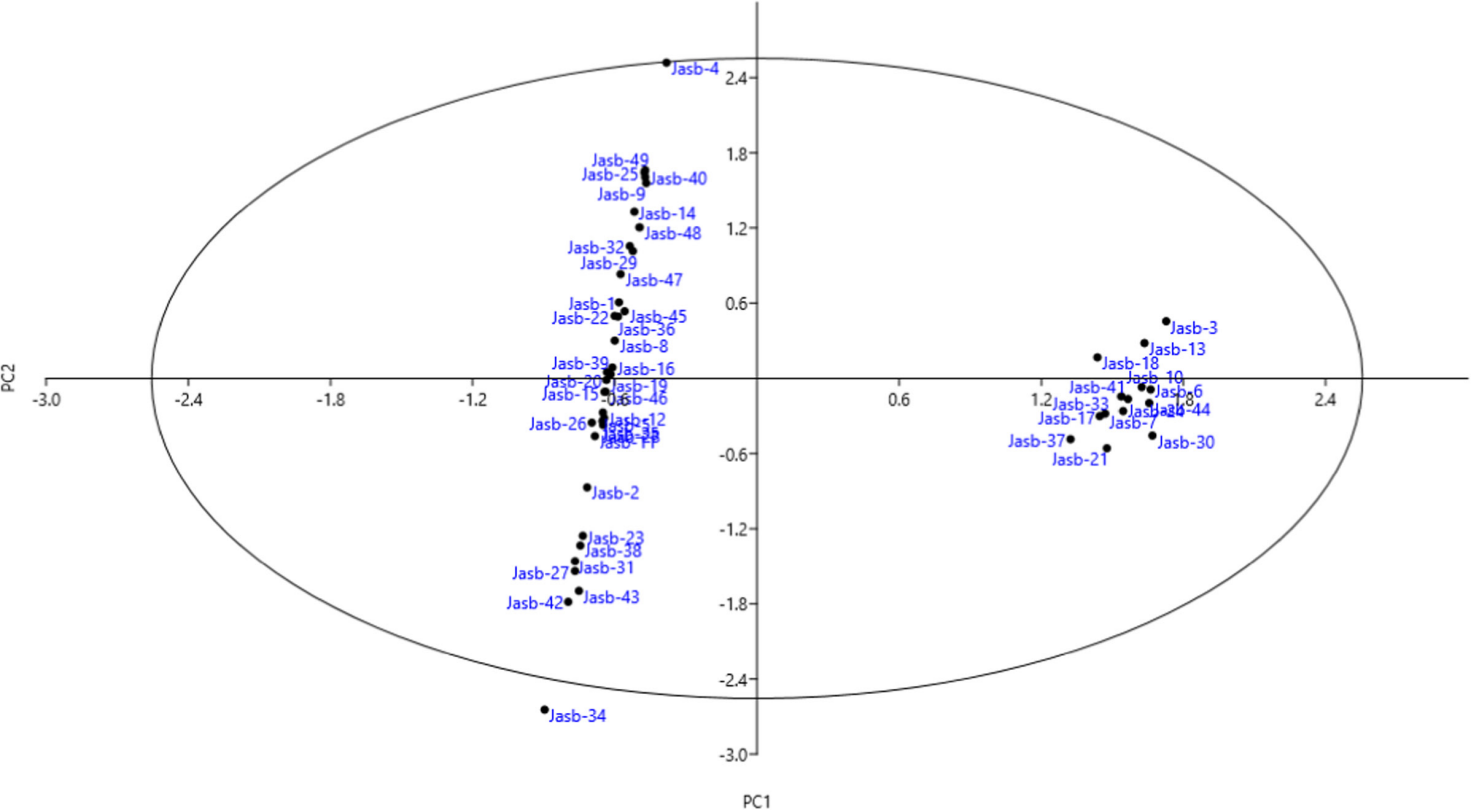
Scatter plot for the studied wild *F. carica* accessions based on PC1/PC2

**FIGURE 3:**
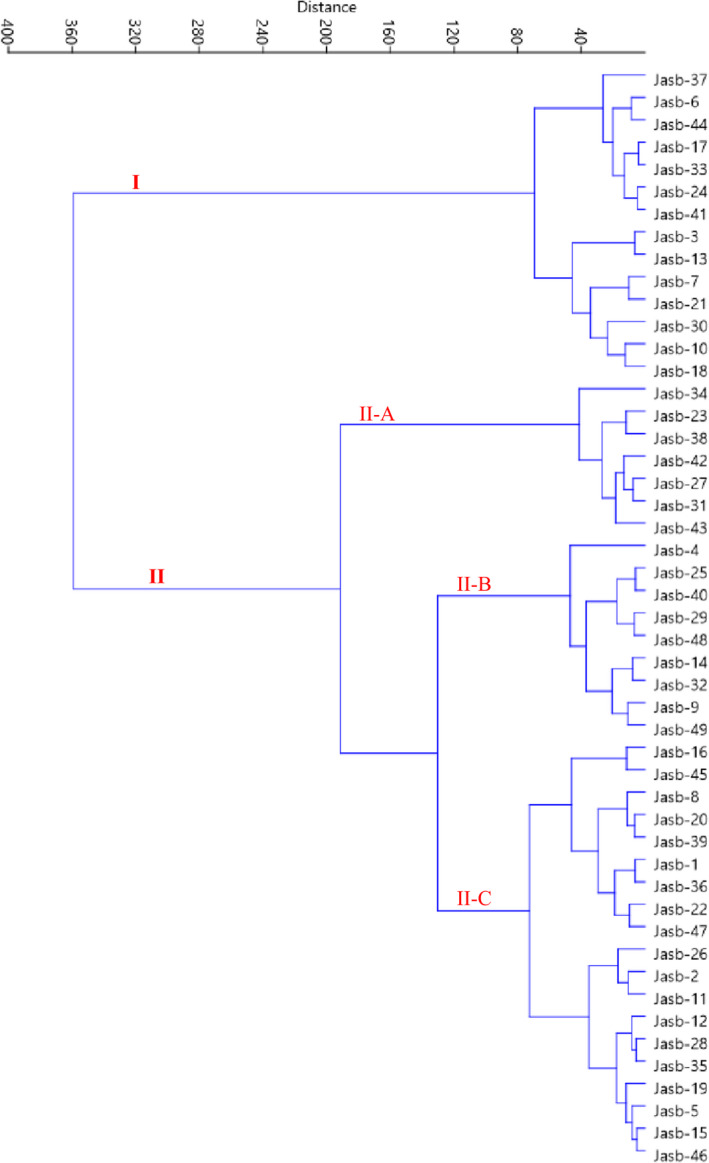
Ward cluster analysis of the studied wild *F. carica* accessions based on morphological traits using Euclidean distances

Based on the scatter plot generated using PC1 and PC2 (33.25% of total variance), the accessions were placed into four groups and most of them were placed into the left side of the plot (Figure 2). Ward dendrogram created according to the data obtained revealed the variation among the accessions and showed two major clusters (Figure 3). The first cluster (I) contained 14 accessions, while the rest of the accessions were placed into the second cluster, forming three subclusters. Subcluster II‐A consisted of seven accessions, subcluster II‐B included nine accessions, while subcluster II‐C contained 19 accessions. The present results showed that the studied accessions had remarkable phenotypic variation, and among them, some accessions with high‐quality fruits in size, color, and taste can be planted and then used in the breeding programs.

The high phenotypic variation was revealed within the fig germplasm studied, in agreement with several studies carried out in different countries such as Turkey (Caliskan et al., 2017), Tunisia (Essid et al., 2017; Gaaliche et al., 2011; Mars et al., 1998), Iran (Khadivi et al., 2018; Mahdavian et al., 2006), Spain (Sanches et al., 2002), Lebanon (Chalack et al., 2005), Jordan (Almajalia et al., 2012), and Morocco (Hssaini et al., 2020).

## 4 CONCLUSION

Broad phenotypic diversity existed among wild fig accessions studied. High level of variability obtained by the studied fig germplasm can be exploited in breeding programs for *F. carica* improvement. Many traits recorded are with high economic importance and consequently usually serve as target traits for selection by fig growers and breeders. Information on the current levels of genetic diversity of germplasm is essential for devising strategies for wild forms conservation. Also, the findings have important implications for fig management to maintain longevity and diversity of the species and will facilitate its use in breeding programs.

## CONFLICT OF INTEREST

The authors declare no conflict of interest.

## RESEARCH INVOLVING HUMAN PARTICIPANTS AND/OR ANIMALS

None.

## INFORMED CONSENT

None.

## Data Availability

The data that support the findings of this study are available from the corresponding author upon reasonable request.
